# Compton profile of few-layer graphene investigated by electron energy-loss spectroscopy

**DOI:** 10.1038/s41598-019-53928-2

**Published:** 2019-11-21

**Authors:** Zhenbao Feng, Xiaoyan Zhang, Yoshiharu Sakurai, Zongliang Wang, Hefu Li, Haiquan Hu

**Affiliations:** 1 School of Physics Science and Information Technology, Shandong Key Laboratory of Optical Communication Science and Technology, Liaocheng University, Liaocheng, 252059 China; 2 Japan Synchrotron Radiation Research Institute, SPring-8, 1-1-1 Kouto, Sayo, Hyogo 679-5198 Japan

**Keywords:** Graphene, Electronic properties and materials

## Abstract

In this paper, acquisition of the valence Compton profile of few-layer graphene using electron energy-loss spectroscopy at large scattering angle is reported. The experimental Compton profile is compared with the corresponding theoretical profile, calculated using the full-potential linearized augmented plane wave method based on the local-density approximation. Good agreement exists between the theoretical calculation and experiment. The graphene profile indicates a substantially greater delocalization of the ground state charge density compared to that of graphite.

## Introduction

Electron energy-loss spectroscopy (EELS) is a powerful tool to study the electronic structure properties of solids^1^. The quantity measured in an EELS experiment is the double differential scattering cross-section^2^

1 $$\frac{{d}^{2}\sigma }{d\varOmega dE}=\frac{4{\gamma }^{2}R}{E{q}^{2}}\frac{k}{{k}_{0}}\frac{df}{dE}(q,E),$$

where γ is the relativistic factor; *R* is the Rydberg energy; *k*_0_ and *k* are wavevectors of the fast electron before and after scattering, respectively; d*f/*d*E* is the generalized oscillator strength (GOS) that is a function of the momentum transfer **q** and the energy loss *E*. The physical information of the GOS depends on the **q** and *E* regimes^2–5^. For small *q*, the GOS contains information about the unoccupied density of states: On the one hand, when $q\cdot r\ll 1$, where **r** represents the electron orbital radius, the dipole-allowed transitions dominate the energy-loss spectrum. On the other hand, when $q\cdot r\ge 1$, monopolar and higher-order transitions become important. The EELS recording in this momentum transfer regime is known as the electron energy-loss near-edge structure (ELNES). For large momentum transfer relative to the inverse electronic orbital size, and for large energy loss (transfer) relative to the binding energy of an electron; the impulse approximation is valid^6,7^. The GOS provides information about the ground-state electron momentum density, known as electron Compton scattering from solids (ECOSS)^8^. The EELS is acquired by tilting the incident beam through a few degrees (~5°) for ECOSS measurement so that only large-angle-scattered electrons are collected^9,10^. The ECOSS technique has proven to be an important alternative for experimentally investigating the electron momentum density distribution of nanomaterials^11,12^.

The past few years have seen a prodigious amount of research on nanocarbon materials, and on graphene in particular^13–15^. In general, understanding the many fascinating properties of graphene requires a detailed knowledge of its electronic structure. Although sufficient work is available regarding the structural, electronic and optical properties of graphene, the experiment and theoretical electron momentum density of graphene still remains unexplored^16–21^.

Compton scattering is a well-established and unique tool to study the electronic structure of materials via their electron momentum density^22–26^. The shape of the Compton profile is sensitive to the state of the valence electrons of the atoms which combine to form the condensed phase of the material^7^. It offers a straight way to test the band structure calculations for different materials^23,25^. Since most of the theoretical studies of the electronic structure of graphene have been carried out using the density functional theory (DFT) and the local density approximation (LDA), it is important to test the applicability of these approaches. In this work, the Compton scattering experiments on graphene are reported using the ECOSS technique. The valence Compton profile of graphene is obtained by subtracting the theoretical core-electron Compton scattering from the experimental profile. In addition, we calculated the valence Compton profile of graphene using a full-potential linearized augmented plane wave (FLAPW) within the framework of the density functional theory^27^. The experimental results are in excellent agreement with the theoretical Compton profile of graphene. Further, the graphene Compton profile is compared with that of graphite both experimentally and theoretically, where a reasonable agreement between experiment and theory was achieved.

## Results and Discussion

**Figure 1:**
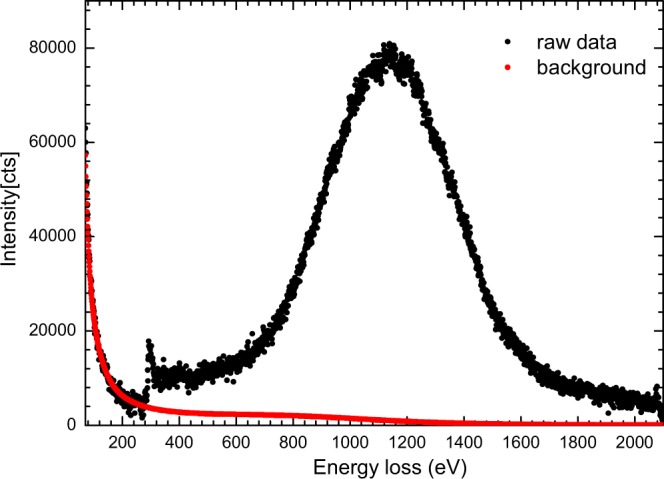
Electron energy-loss spectrum (EELS) of few-layer graphene at ~73 mrad scattering angle with a 30 s exposure time (black dots), along with the simulated elastic-inelastic scattering background (red dots).

**Figure 2:**
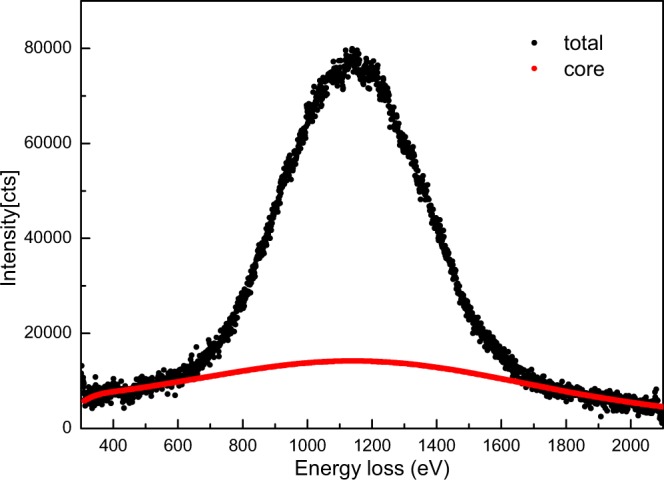
Measured EELS after background subtraction (black dots) and the contribution from the core Compton scattering as determined by Hartree-Slater method (red dots).

The raw electron Compton scattering spectrum of graphene by recording EELS at the scattering angle of ~73 mrad is shown in Fig. 1. It is worth pointing out that the directions of momentum transfers predominantly lay in the basal plan at this large scattering angle. We thus predominantly probed the basal plan Compton profile of graphene in this experiment. The Compton scattering spectrum extends over hundreds of electron volts, so the typical power-law background subtraction method does not work. In this work, the background of the ECOSS spectrum was subtracted by parameterized simulations of combined elastic and inelastic scattering events^28^. A characterization angle of 15 mrad and the ratio of quasi-elastic to inelastic scattering cross-sections of 0.8 were chosen for the background simulation. The core Compton scattering spectrum as a function of energy loss (eV) was determined using the Hartree-Slater method at a momentum transfer of 9.25 a.u., as shown in Fig. 2. We note that the theoretical core Compton scattering spectrum is in good agreement with the experimental profile at both the leading and trailing edges. This shows the rationality of our background subtraction^29^.

**Figure 3:**
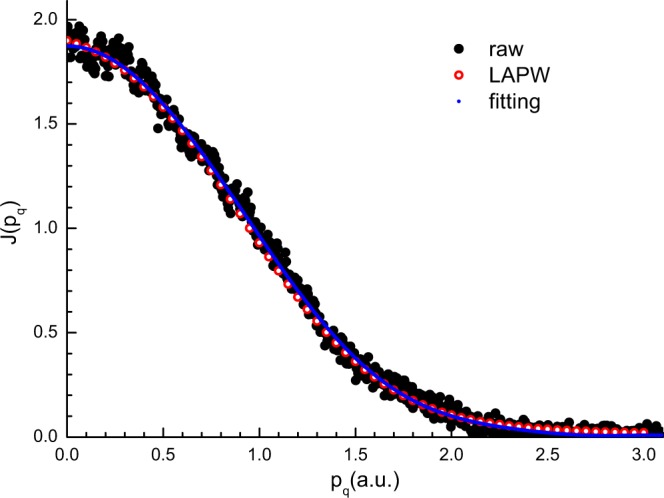
Experimental valence Compton profile of few-layer graphene obtained by the TEM (black dots), the polynomial fitting of the experimental data (blue line), and the FLAPW calculated results (red line).

**Figure 4:**
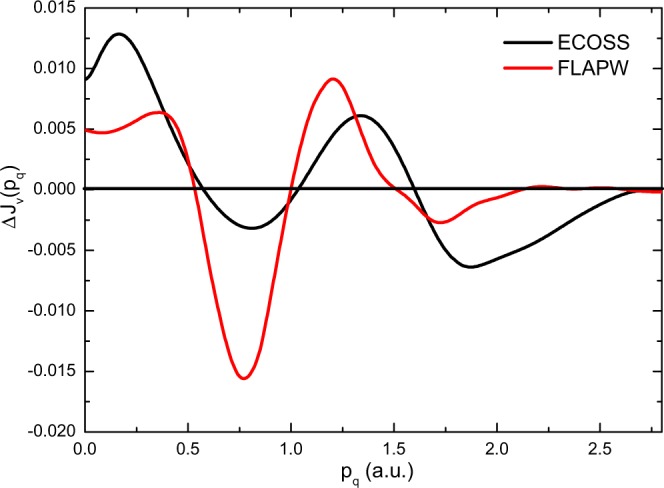
Comparison of the Compton profile difference for graphene and graphite, $\Delta {J}_{{\rm{v}}}({p}_{q})={J}_{{\rm{v}}}^{{\rm{graphene}}}({p}_{q})-{J}_{{\rm{v}}}^{{\rm{graphite}}}({p}_{q})$, obtained by ECOSS experiment and FLAPW calculations.

Figure 3 shows the measured and calculated valence Compton profiles (${J}_{{\rm{v}}}({p}_{q})$) of graphene as a function of momentum (*p*_*q*_). The theoretical profile was convolved with the experimental resolution function. Details of our experimental data processing procedure have been previously described^30^. The Compton profile of graphene was calculated as a longer lattice constant along the *c*-axis (20 Å), while graphite was 6.77 Å. In order to compare experimental results with our calculations, the theoretical in-plane profile was obtained by averaging the Compton profiles calculated for $[10\bar{1}0]$ and $[11\bar{2}0]$ directions. The excellent agreement between the calculated and measured profiles is evident in the Fig. 3. Compton profile differences are useful for comparing experiment and theory^7,31^. The experimental Compton profile of graphite was obtained under the same experimental conditions as that of graphene by ECOSS technique^30,32^. Figure 4 plots the differences ($\Delta {J}_{{\rm{v}}}({p}_{q})$) between the graphene (${J}_{{\rm{v}}}^{{\rm{grephene}}}({p}_{q})$) and graphite (${J}_{{\rm{v}}}^{{\rm{graphite}}}({p}_{q})$) profiles for both the FLAPW theoretical calculations and the ECOSS measurements. Good agreement is found in the Fig. 4 between the FLAPW calculated differences and the ECOSS measured differences, where the experimental and calculated $\Delta {J}_{{\rm{v}}}({p}_{q})$ exhibit similar trends. The dip near *p*_*q*_ = 0.75 a.u. may arise from π-bonding anisotropy in graphite^33^. The observed narrowness of the graphene Compton profile with respect to that of graphite can be interpreted as a larger delocalization of valence electrons in the graphene than in graphite. In graphite, the electrons form π bonds between two layers via the weak van der Walls forces. In graphene, however, these π bonds are incomplete and the electrons forming such bonds behave almost as delocalized electrons, associated with the curving graphene layer by means of dangling bonds.

## Conclusions

To summarize, the valence Compton profile of few-layer graphene was obtained by measuring the EELS in a TEM and comparing it with the FLAPW calculation based on the local-density approximation band theory framework. The experimental results are in good agreement with the theoretical calculations. The differences between the Compton profiles of graphene and graphite obtained both experimentally and via FLAPW calculations showed good agreement. Both the experimental and theoretical results indicate that the ground-state electronic density is more delocalization in graphene than in graphite.

## Experimental

The experiments were performed with a transmission electron microscope (TEM; FEI TECNAI F20, Thermo Fisher Scientific) equipped with an imaging filter (Gatan Tridiem), where the TEM was operated at an accelerating voltage of 120 kV. Commercially-available 300-mesh copper TEM grids with amorphous holey carbon film supporting transferred graphene (Graphene Supermarket, https://graphene-supermarket.com) were used, where the graphene was produced by chemical vapor deposition and consisted of 1–6 layers. The EELS technique was used to measure the electron Compton scattering of the few-layer graphene film suspended over the TEM holey carbon film holes, where the TEM was in the diffraction mode with a parallel incident beam. Though the operating energy of the incident beam was well above the knock-on threshold of graphene, the dose was spread over a sufficiently large area so that the electron beam damage to the samples was not expected to be significant over the short acquisition times (30 s) used herein^34^. The camera length was 300 mm and the spectrometer entrance was set to 2.5 mm. Further, the collection semi-angle was 2.44 mrad and the corresponding momentum transfer uncertainty was 0.16 a.u. The energy resolution was determined from the full width at half maximum of the zero-loss peak at 3 eV, which when converted to a momentum resolution was found to be 0.012 a.u. The total momentum resolution was estimated to be on the order of 0.1 a.u.^10^. To reduce the statistical error, more than five spectra were acquired from the sample, whereupon additional measurements were obtained to determine morphological variations on the sample to ensure that serious radiation damage had not occurred.
